# Clinical Characteristics and Prognostic Significance of TERT Promoter Mutations in Cancer: A Cohort Study and a Meta-Analysis

**DOI:** 10.1371/journal.pone.0146803

**Published:** 2016-01-22

**Authors:** Ping Yuan, Jin-lin Cao, Abudumailamu Abuduwufuer, Lu-Ming Wang, Xiao-Shuai Yuan, Wang Lv, Jian Hu

**Affiliations:** Department of Thoracic Surgery, The First Affiliated Hospital, School of Medicine, Zhejiang University, Hangzhou, China, 310003; CNR, ITALY

## Abstract

**Background:**

The prevalence of telomerase reverse transcriptase (TERT) promoter mutations (pTERTm) in non-small-cell lung cancer (NSCLC) have been investigated, but the results were inconsistent. In addition, several studies have analysed the role of pTERTm in the etiology of various types of cancers, however, the results also remain inconsistent.

**Methods:**

The genomic DNA sequence of 103 NSCLC samples were analysed to investigate the frequency of pTERTm in these patients and to establish whether these mutations are associated with their clinical data. Furthermore, a meta-analysis based on previously published articles and our cohort study was performed to investigate the association of pTERTm with patient gender, age at diagnosis, metastasis status, tumour stage and cancer prognosis (5-year overall survival rate).

**Results:**

In the cohort study, 4 patients had C228T and 2 had C250T, with a total mutation frequency up to 5.8%. Significant difference of clinical data between pTERTm carriers and noncarriers was only found in age at diagnosis. In the meta-analysis, We found that pTERTm carriers in cancer patients are older than noncarriers (Mean difference (MD) = 5.24; 95% confidence interval [CI], 2.00 to 8.48), male patients were more likely to harbour pTERTm (odds Ratios (OR) = 1.38; 95% CI, 1.22 to 1.58), and that pTERTm had a significant association with distant metastasis (OR = 3.78; 95% CI, 2.45 to 5.82), a higher tumour grade in patients with glioma (WHO grade III, IV vs. I, II: OR, 2.41; 95% CI, 1.88 to 3.08) and a higher tumour stage in other types of cancer (III, IV vs. I, II: OR, 2.48; 95% CI, 1.48 to 4.15). pTERTm was also significantly associated with a greater risk of death (hazard ratio = 1.71; 95% CI, 1.41 to 2.08).

**Conclusions:**

pTERTm are a moderately prevalent genetic event in NSCLC. The current meta-analysis indicates that pTERTm is associated with patient age, gender and distant metastasis. It may serves as an adverse prognostic factor in individuals with cancers.

## Introduction

The telomerase reverse transcriptase (TERT) gene encodes a highly specific reverse transcriptase that adds repeats to the 3′ end of chromosomes [1]. The increased telomerase activity allows tumours to avoid the induction of senescence by the preservation of their telomere ends [2,3]. The promoter region of TERT is considered to be the most imperative regulatory element for telomerase expression; it contains several binding sites for factors that regulate gene transcription [4]. Inhibition of telomerase activity for reversion of the immortal phenotype of tumour cells has been one of the most common approaches for cancer therapy [5]. Recent studies have demonstrated that activation of telomerase via transcriptional TERT unregulation can be caused by mutation in the core promoter region of TERT (chr5:1,295,228C>T [C228T], chr5:1,295,250C>T [C250T], et al.) [6,7]. These mutations confer 2-fold to 4-fold increased TERT transcriptional activities by the creation of binding sites for ETS/ternary complex factors (TCF) transcription factors and then upregulate TERT expression, suggesting a potential mechanism for telomerase activation in tumourigenesis [7,8].

The relative characteristics and prognostic effects of TERT promoter mutation (pTERTm) on carriers and noncarriers with cancer are unclear. Statistical difference in gender distribution between pTERTm carriers and noncarriers was found in some studies that male cancer patients are more likely to harbour pTERTm [9,10,11]. Recently, Gandolfi and Wang reported that pTERTm are associated with distant metastases in upper tract urothelial carcinoma and papillary thyroid cancer. Such association of pTERTm may also present in other cancers. In addition, the effects of pTERTm on patient outcome are obscured. Several studies have demonstrated a less favourable prognosis of glioma in pTERTm carriers than in noncarriers [12,13,14,15,16,17], whereas a recent report found a better outcome for pTERTm carriers [18].

The prevalence and association of pTERTms with non-small-cell-lung-cancer (NSCLC) patients have been studied but showed different results. Ma and colleagues found a proportion of 2.67% NSCLC patients in their cohort had pTERTm [19], whereas other studies failed to detect pTERTm [20,21,22]. By conducting a cohort study in NSCLC patients and a meta-analysis, we have attempted to further strengthen the prevalence of pTERTm in NSCLC and to provide definitive evidence of the relative effectiveness and characteristics of pTERTm in cancer patients. This is the first meta-analysis to evaluate the association of pTERTm with cancer. The results could provide insight into the biology of pTERTm, to understand the clinical prognosis of these mutation carriers and to offer implications for the design of clinical trials, particularly those of anticancer agents that target the TERT.

## Methods

### Cohort study

#### Patients and tissue samples

We obtained 103 liquid nitrogen–stored tissue samples of 103 NSCLC patients with pathologic confirmation who were admitted to the First affiliated Hospital of Zhejiang University between 2013 and 2014. Sufficient high-quality tumour samples were taken at the time of surgical resection by well-trained physicians with written informed consent from each patient. Each sample was placed in liquid nitrogen immediately after resected and stored in -80°C refrigerator. Patient clinical data were collected and their information was anonymized and de-identified prior to this analysis. This cohort study was conducted under the approval of the Ethics Committees of the First affiliated Hospital of Zhejiang University

#### DNA extraction and mutation analysis

DNA extraction and polymerase chain reaction amplification for sequencing of the TERT promoter were performed in all cases by standard protocols. The genomic DNA of tumour tissue was extracted with a QiAamp DNA Mini Kit (Qiagen, Hilden, Germany) and purified with an EZNA MicroElute DNA Clean-Up kit (OMEGA). Polymerase chain reaction (PCR) amplification of the TERT promoter region covering the mutations (from –27 to –286) was performed using primers: 5′ CCC ACG TGC GCA GCA GGA C3′ (forward) and 5′ CTC CCA GTG GAT TCG CGG GC3′ (reverse), With 3 minutes at 95°C; 35 cycles at 95°C 15 seconds, 63°C 15 seconds, 72°C 1 minute, followed by a final step at 72°C for 5 minutes. After gel electrophoresis to confirm the quality of the PCR products, sequencing PCR was performed using a Big Dye terminator version 3.1 cycle sequencing ready reaction kit (Applied Biosystems), and DNA sequence was analysed on an ABI PRISM 3730 automated genetic analyser (Applied Biosystems), All samples were checked in forward and reverse directions.

#### Statistical method of cohort study

Statistical analyses were carried out using the SPSS16.0 software package. Associations between pTERTm and the patients’ categorical variables were analysed with a chi-square test, Continuous data were summarised as the mean ± SD and analysed with the Mann-Whitney Wilcoxon test. Values of p less than 0.05 were considered significant.

### Meta-analysis

#### Literature search

We searched PubMed and Web of Science for articles published before March 2015, using the systemic literature search terms “telomerase reverse transcriptase”, “promoter”, and “mutation”. The reference lists of the articles retrieved were further screened for other potential studies. We made every attempt to obtain the necessary information from the first and corresponding authors by e-mail if insufficient data were reported in the article (i.e., missing data, missing Kaplan-Meier curves or any other uncertainties).

#### Inclusion and exclusion criteria

All of the studies included in this meta-analysis met the following criteria: (a) articles about the pTERTm and human cancer that were published in English. (b) availability of detailed genotype data or frequencies that could be calculated from the article text; (c) sufficient data to calculate an odds ratio (OR) or hazard ratio (HR, for prognosis analysis) with a 95% confidence interval (CI); (d) if survival data is not available for calculating HR, survival curves for pTERTm carriers and noncarriers is necessary. The exclusion criteria were: (a) published as an abstract, case report, comment letter, review or editorial; (b) non-human studies; (c) duplicate studies, in which case the latest or largest study were included.

#### Data extraction

Two reviewers independently assessed all of the potentially relevant studies and reached a consensus on all of the items. Any disagreements were reconciled by discussion and consensus. The following data were collected from each study: first author, year of publication, type of cancer, population, sequencing method and the number of carriers and noncarriers.

#### Quality assessment

The quality of the studies included was evaluated according to the Newcastle-Ottawa scale (NOS) quality assessment, which is available at http://www.ohri.ca/programs/clinical_epidemiology/oxford.asp. This evaluation system focuses on three aspects of a study (selection of patients, comparability of baseline characteristics and outcome assessment). The quality of the study was denoted by a numerical score from 0 to 9, with 9 representing the highest quality. Quality assessment was conducted by two independent reviewers. The original papers were scanned when disagreements occurred. Unsettled disagreements were referred to a third researcher for a final decision.

#### Statistical method of meta-analyses

The meta-analyses, subgroup analyses and sensitivity analyses were performed with Review Manager (revman) version 5.1 software. The meta-regression, Begg’s and Egger’s test were performed with STATA software (version 12.0 Stata Corp LP, College Station, Texas).

For dichotomous outcomes, Odds Ratio (OR) with 95% confidence intervals was calculated by using a fixed effect model (Mantel-Haenszel method) [23] for P_*Heterogeneity*_ > 0.05, or random effect model (DerSimonian and Laird method) [24] for P_*Heterogeneity*_ < 0.05. Such as the assessment of association between pTERTm and gender (male vs. female), lymphatic metastasis (positive vs. negative), distant metastasis (positive vs. negative), tumour stage (III/IV vs. I/II), and Glioma WHO grade (III/IV vs. I/II). The dependent variables in these studies are the frequencies of event versus non-events. The significance of the combined OR was determined with a Z test, in which p < 0.05 was considered statistically significant. For continuous outcomes, the mean difference (MD) was calculated based on the mean and standard deviation given in the included studies. So the association between pTERTm and patient age at diagnosis was evaluated by mean age difference (carriers vs. noncarriers) combined with the corresponding 95% CIs. Pooled HR with a 95% confidence interval was calculated for the association between 5-year overall survival and pTERTm status (carriers vs. noncarriers). HR < 1 means that the prognosis of patients of pTERTm carrier is worse than non-carriers, while HR > 1 means the opposite. If a direct report of survival were not available, then the survival data read from Kaplan-Meier curves were read by Engauge Digitizer version 4.1 (http://digitizer.sourceforge.net/). Population data sets were categorized as Asian and non-Asian. Stratified analyses were performed by cancer type (If a cancer type contained only one data source, it was combined into the “other cancers” group.). The evaluation of the meta-analysis results included an examination of the heterogeneity, an analysis of the sensitivity, meta-regression and an examination for publication bias.

The heterogeneity between studies was evaluated using a chi-square–based Q test and a p value of less than 0.05 was considered statistically significant. The Higgins I^2^ was calculated to quantitatively estimate the heterogeneity, with I^2^ < 25%, I^2^ = 25–75% and I^2^ > 75% representing low, moderate and high heterogeneity, respectively. Subgroup and meta-regression were conducted to delineate the major sources of heterogeneity. Sensitivity analyses were performed to assess the stability of the results and to identify the individual potential influences on the OR or HR. Funnel plots and Egger’s test were used for the diagnosis of potential publication bias, An asymmetric plot suggests a possible publication bias and the P value of Egger’s test being considered representative of significant publication bias if it was less than 0.05.

## Results

### Results of the cohort study

The study included 103 surgical specimens from patients with NSCLC. The results of the cohort study are shown in Table 1. We identified six mutations (5.8%) in the TERT promoter region (four C228Ts and two C250Ts) (Table 2). The associations of the patient characteristics and clinical features with pTERTm status amongst our patients showed a statistically significant difference only for age. The pTERTm carriers tended to be older at the time of diagnosis than the noncarriers (p = 0.031). No significant differences were found in the distributions of gender (P = 0.551), tumour size (0.196), lymphatic metastasis (p = 0.567), distant metastasis (p = 0.654), tumour stage (p = 0.6) or other clinical features (Table 1).

**Table 1:** Results of association of pTERTm with NSCLC patient characteristics in the cohort study.

		pTERTm		
Characters	All Cases	Non-carriers	Carriers	P value
**Total**	103	97	6	
**Age at diagnosis**				0.031
Mean ± SD	61.4 ± 9.2	61.0 ± 8.8	69.2 ± 9.7	
**Gender**				0.551
Male	58	54	4	
Female	45	43	2	
**Smoking history**				0.826
Smoker	47	44	3	
Never smoke	56	53	3	
**Tumour size (cm)**				0.196
Mean ± SD	3.07 ± 1.82	3.01 ± 1.74	4 ± 2.79	
**Tumour Grade (n = 95)**				0.503
I/II	44	42	2	
III	51	47	4	
**Lymphatic metastasis**				0.567
Positive	23	21	2	
Negative	80	76	4	
**Distant metastasis**				0.654
Positive	2	2	0	
Negative	101	95	6	
**Pathologic stage**				0.600
I/II	79	75	4	
III/IV	24	22	2	
**pathologic T stage**				0.449
T1/T2	84	80	4	
T3/T4	19	17	2	
**Histology**				
ADC	68	66	2	
SCC	31	27	4	
ASC	4	4	0	

ADC: adenocarcinoma; SCC: squamous cell carcinoma; ASC: adenosquamous carcinoma; pTERTm: TERT promoter mutation

**Table 2:** Clinicopathologic details of 6 NSCLC patients with TERT promoter mutation.

Gender	AAD	Smoker	Tumor size (cm)	Tumor grade	Lymph node status	Distant metastasis	pathologic stage	T stage	Histology	TERT promoter mutation
Male	62	Yes	2.5	2	N0	M0	Ia	T1b	SCC	C228T
Female	87	No	2	3	N0	M0	Ib	T2a	ADC	C250T
Female	69	No	2.5	3	N2	M0	IIIa	T1b	ADC	C228T
Male	60	No	3.5	3	N2	M0	IIIa	T2a	SCC	C228T
Male	66	Yes	4	2	N0	M0	IIb	T3	SCC	C228T
Male	71	Yes	9.5	3	N0	M0	IIb	T3	SCC	C250T

AAD: age at diagnosis

### Results of the meta-analysis

#### Characteristics of the identified studies

**Fig 1:**
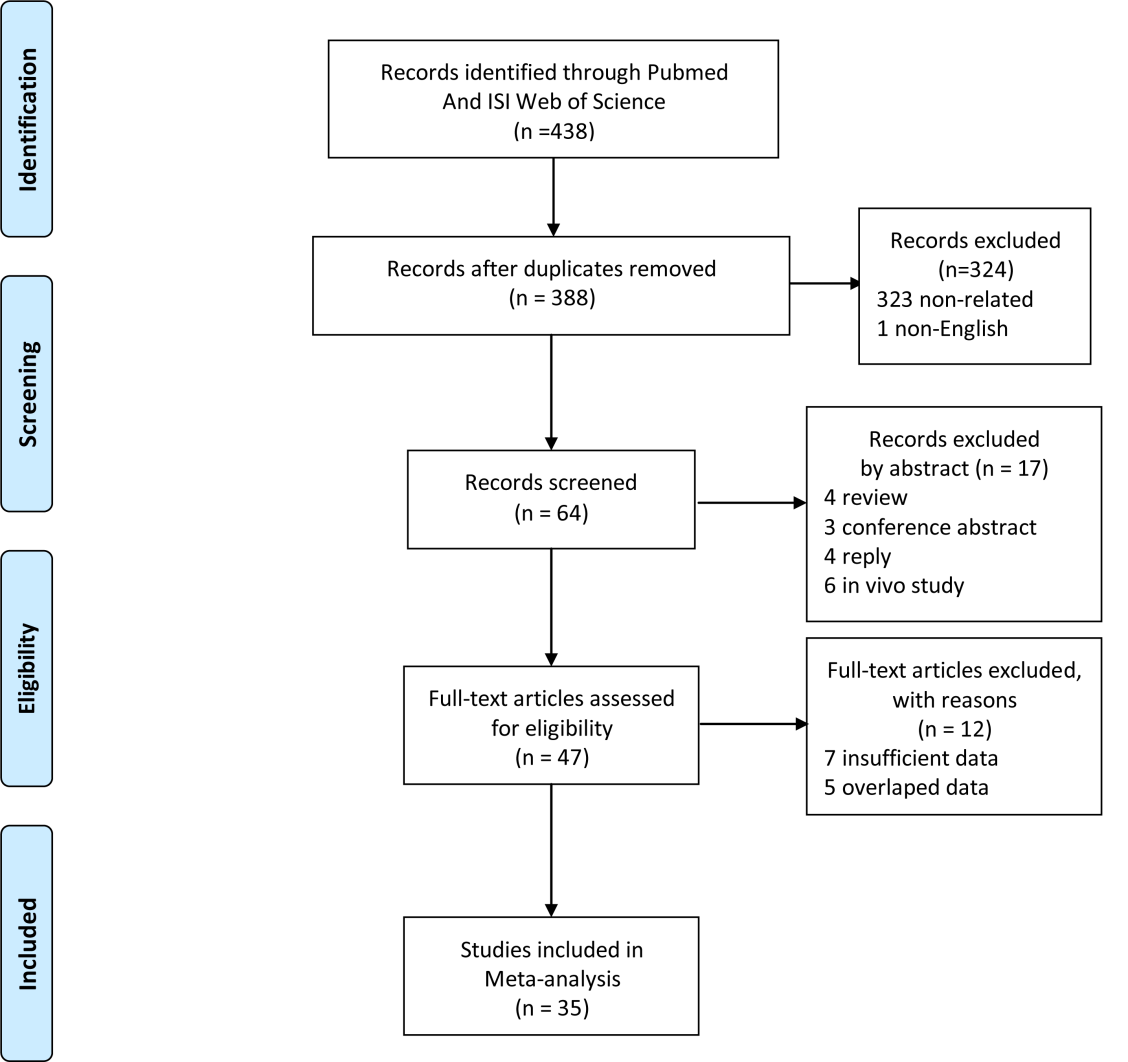
PRISMA 2009 Flow Diagram. A list of full-text excluded articles.

The detailed selection process is demonstrated in Fig 1. In the initial search, 245 studies were found in PubMed, 193 studies were found in Web of science. A total of 388 studies remained after the initial elimination for duplication. 341 studies were excluded after the titles and abstracts were examined. Following a full text review and detailed evaluation, 35 articles were included in our analyses (Table 3). Each study was published between 2013 and 2015 by authors from China, Korea, Japan, Austria, The United States, Germany, Italy, France, Sweden and Portugal. Among the 35 studies, Nine studies assessed glioma [12,13,18,25,26,27,28,29,30], seven studies assessed thyroid cancer [9,14,31,32,33,34,35], five studies assessed melanoma[10,15,16,36,37], two studies each assessed bladder cancer [38,39], renal cell carcinoma [40,41] gynecologic cancer [42,43], hepatocellular carcinoma [11,44] and urothelial carcinoma [17,45]. One study each assessed lung cancer [19], adrenal cancer [46] laryngeal cancer [47] and meningioma [48]. The results of our cohort study (Yuan P) are also included in this meta-analysis. Thus, 36 studies with 3001 carriers and 8384 noncarriers were analysed. In addition, in that some independent variables are not available in certain articles, the numbers of studies in different analyses are varied.

**Table 3:** Basic characters of included studies.

Study/year	Population	carriers/ total	Mean age	Primary treatment	FU date, (month)	Sequencing method	Period	NOS
**Glioma**								
Spiegl-Kreinecker/2015	Austria	92/126	60	S/C/R	mean>12	Sanger	1998–2013	7
Chen, A K/2014	China	67/237	40.5	S/R/C	mean:113	Sanger	1990–2012	8
Chen, C/2014	China	45/101	47.0	S/R/C	mean>12	Sanger	2006–2007	7
Killela, P J/2014	USA	281/473	55.1	S	mean>60	Sanger	NR	7
Labussiere,M/2014	France	491/807	46.1	S	mean:18	Sanger	NR	8
Remke,M/2014	Multi-center (non-Asian)	96/466	10.1	S	median:44	Sanger	NR	5
Simon, M/2014	Germany	143/178	60.9	S/C	mean:17	Sanger	1995–2002	8
Park, C K/2014	Korea	29/48	48.5	S/C	12<mean<60	Sanger	NR	6
Arita, H/2013	Multi-center (non-Asian)	43/88	52.9	S	NR	Pyrosequencing	NR	5
**Thyroid cancer**								
Muzza, M/2015	Italy	30/240	48.8	S	mean: 78.9	sanger	NR	9
Gandolfi, G/2015	Italy	21/121	48.06	S	mean:124.1	Sanger	1979–2013	9
Liu, T/2014	Sweden	31/107	55.9	S	mean:122	Sanger	NR	9
Melo, M/2014	Multi-center (non-Asian)	58/411	48.2	S/R	mean:93.6	Sanger	NR	8
Wang, N/2014	Sweden	4/63	48.9	S	mean:118	Sanger	1986–2004	9
Xing, M/2014	USA	61/507	45.9	S/R	mean:38.7	Sanger	1990–2012	7
Liu, X/2014	Multi-center (Asian)	108/430	44.6	S	NR	Sanger	NR	5
**Melanoma**								
Egberts, F/2014	Germany	33/92	48.1	S	mean>60	pyrosequencing	1998–2011	7
Griewank, K G/2014	Multi-center (non-Asian)	154/362	52.0	NR	median:35	Sanger	NR	7
Populo, H/2014	Portugal	26/116	59.0	R	mean:48	BigDye Terminator	2009–2013	8
Xie, H/2014	Multi-center (mixed)	4/35	79.8	NR	mean:135	Sanger	NR	7
Heidenreich, B/2014	Spain	109/287	NR	NR	NR	Sanger	2000–2012	5
**Lung cancer**								
Ma, X	China	12/455	60	S	median:12.1	Sanger	2007–2011	5
Yuan, P	China	6/103	61.8	S	mean: 12.1	Sanger	2013–2014	6
**Bladder cancer**								
Rachakonda, P S/2013	Sweden	186/327	71.2	S/R/C	mean:180	Sanger	1995–1996	8
Allory, Y/2014	Multi-center (non-Asian)	361/468	68.1	S/C	mean:53	Sanger	NR	5
**Renal cell carcinoma**								
Hosen, I/2014	Germany	12/188	65	S	mean:121	Sanger	NR	8
Wang, K/2014	China	9/96	54.5	S	NR	Sanger	NR	6
**Gynecologic cancer**								
Huang, H N/2014	China	12/70	48	S/C	mean:31	Sanger	1995–2001	7
Wu, R C/2014	Multi-center (mixed)	37/233	51.8	S	NR	Sanger	NR	7
**Hepatocellular carcinoma**								
Chen, Y L/2014	China	57/195	56.6	S/C	mean:96	Sanger	1983–1997	8
Nault, J C/2014	France	179/305	58.6	S	mean:123	Sanger	1997–2004	5
**Urothelial carcinoma**								
Wu, S/2014	China	120/216	62.1	S	mean:120	Sanger	NR	7
Kinde, I/2013	USA	9/78	54.5	S	mean:38	Safe-SeqS	2000–2012	7
**Laryngeal cancer**								
Qu, Y/2014	China	64/235	60.0	S	median:38	Sanger/pyrosequencing	NR	8
**Meningioma**								
Goutagny, S/2014	France	6/73	51.3	S	mean:122	Sanger	NR	5
**Adrenal cancer**								
Liu, T/2014	Multi-center (non-Asian)	5/47	52.9	S	mean:86	Sanger	NR	7

FU date: follow-up date; NOS: Newcastle-Ottawa scale; NR: no report.

#### Association of pTERTm with Patient age, gender, metastasis status and tumour stage

The overall results show that pTERTm carriers were older than noncarriers (MD = 5.24; p < 0.001) from a random model. Stratification analysis decreased heterogeneity and identified increased MD in subgroup of glioma and lung cancer, whereas melanoma displayed a reversed pattern (MD = -5.74; p = 0.02). No significant difference was found in other cancers. (Table 4, S1 Fig)

**Table 4:** Results of Meta-analyses Stratified by cancer type.

				MD, 95% CI			Heterogeneity	
Analyses	No. study	Total No. carriers	Noncarriers	Fixed effect model	Random effect model	p	I^2^ (%)	P
**Age (carriers vs. noncarriers)**	**26**	**1352**	**3756**	- -	**5.24 [2.00, 8.48]**	**0.002**	**92**	**<0.001**
Glioma	4	260	155	10.69 [8.51, 12.87]	- -	<0.0001	50	0.11
Thyroid cancer	7	313	1566	- -	12.17 [8.70, 15.64]	<0.0001	67	0.006
Melanoma	4	293	508	-5.74 [-7.72, -3.77]	- -	0.02	0	0.2
Lung cancer	2	18	540	8.11 [4.73, 11.49]		<0.0001	0	1
Renal cell carcinoma	2	21	263	0.27 [-4.76, 5.30]		0.92	89	0.67
Urothelial cancer	2	129	165		0.61 [-9.55, 10.77]	0.002	93	0.003
Other cancer	5	318	559	- -	0.60 [-6.04, 7.23]	0.02	89	<0.001
		Total No.		OR (95% CI)			Heterogeneity	
Analyses		carriers	Noncarriers	Fixed effect model	Random effect model	p	I^2^ (%)	P
**Gender (Male vs. Female)**	**28**	**1969**	**4472**	**1.38 [1.22, 1.58]**	- -	**<0.0001**	**31**	**0.06**
Glioma	5	414	599	0.95 [0.70, 1.29]	- -	0.73	0	0.69
Thyroid cancer	7	200	1576	2.13 [1.56, 2.91]	- -	<0.0001	32	0.18
Melanoma	5	402	686	1.42 [1.10, 1.82]	- -	0.006	9	0.36
Hepatocellular carcinoma	2	236	264	2.01 [1.26, 3.19]	- -	0.003	65	0.09
Lung cancer	2	18	552	1.06 [0.40, 2.79]	- -	0.91	0	0.58
Renal cell carcinoma	2	21	263	0.96 [0.39, 2.38]	- -	0.93	0	0.8
Other cancer	5	678	532	1.23 [0.95, 1.59]	- -	0.12	0	0.59
**LM (positive vs. negative)**	**11**	**395**	**1793**	- -	**1.02 [0.71, 1.46]**	**0.93**	**53**	**0.02**
Thyroid cancer	5	194	1299	- -	1.17 [0.69, 1.97]	0.56	69	0.01
Other cancer	6	201	494	0.85 [0.58, 1.27]	- -	0.43	0	0.62
**DM (positive vs. negative)**	**14**	**700**	**2353**	- -	**3.78 [2.45, 5.82]**	**<0.0001**	**62**	**0.001**
Thyroid cancer	6	214	1536	4.01 [3.15, 5.10]	- -	<0.0001	21	0.28
Melanoma	2	111	205	5.68 [0.94, 34.41]	- -	0.06	0	0.82
Renal cell carcinoma	2	25	267	- -	4.87 [0.32, 73.98]	0.18	90	0.001
Other cancer	4	350	345	- -	2.44 [0.67, 8.84]	0.25	76	0.005
**Tumor stage (III/IV vs. I/II)**	**15**	**608**	**2756**	- -	**2.48 [1.48, 4.15]**	**0.0005**	**75**	**<0.001**
Thyroid cancer	5	176	1231	- -	5.09 [2.73, 9.49]	<0.0001	64	0.03
Melanoma	3	291	365	- -	2.50 [0.74, 8.42]	0.14	90	<0.001
Lung cancer	2	18	552	1.21 [0.45, 3.27]	- -	0.71	0	0.4
Gynecologic cancer	2	38	193	0.95 [0.43, 2.10]	- -	0.9	0	0.7
Renal cell carcinoma	2	21	263		**2.80 [0.21, 36.72]**	0.43	86	0.007
Laryngeal cancer	1	64	170	- -	0.92 [0.52, 1.64]	0.78	- -	- -
**Glioma WHO grade (III&IV vs. I/II)**	**4**	**722**	**629**	**2.41 [1.88, 3.08]**	- -	**<0.00001**	**0**	**0.41**
		Total No.		HR (95% CI)			Heterogeneity	
Analyses		carriers	Noncarriers	Fixed effect model	Random effect model	p	I^2^ (%)	P
**Prognosis**	**25**	**2179**	**4236**	- -	**1.71 [1.41, 2.08]**	**<0.0001**	**72**	**<0.001**
Giloma	7	898	1752	- -	1.52 [1.14, 2.02]	0.004	70	0.003
Thyroid cancer	5	210	1051	- -	2.73 [1.47, 5.08]	0.002	73	0.005
Melanoma	4	217	392	- -	1.52 [0.83, 2.81]	0.18	75	0.008
Gynecologic cancer	2	49	217	2.08 [1.23, 3.53]	- -	0.006	70	0.07
Bladder cancer	2	547	200	1.21 [0.95, 1.53]	- -	0.13	0	0.64
Other cancer	**5**	**258**	624	1.45 [1.17, 1.78]	- -	0.0005	40	0.16

OR: odds ratio; MD: mean difference; HR: hazard ratio; WHO: World Health Organization; LM: lymphatic metastasis; DM: distant metastasis

We also found that male cancer patients were more likely to harbour pTERTm (OR = 1.38, p < 0.0001). But non-significant risk was found in glioma, lung cancer and renal cell carcinoma (Table 4, S2 Fig). As for lymphatic metastasis, statistical significance was not found, but cancer patients who harboured pTERTm were much more likely to have distant metastasis (OR = 3.78; p < 0.0001) and a higher tumour stage (III/IV vs. I/II: OR = 2.48; p = 0.0005) (Table 4, S3 Fig and S4 Fig). Stratified analyses of distant metastasis and stage performed on cancer types revealed that the significant risk was only observed in thyroid cancer. In addition, an analysis of tumour stage was not available for glioma, but glioma patients with pTERTm were more likely to have a higher WHO grade (III/IV vs. I/II): OR, 2.41; p < 0.0001) (Table 4).

For the overall comparisons, significant heterogeneity was observed except for gender analysis. However, most of the heterogeneity decreased markedly or disappeared after stratification, excepted for “other cancer” in age analysis, renal cell carcinoma in distant metastasis and melanoma in stage analysis (I^2^ > 75). Sensitivity analysis with one study omitted each time showed that the significance of the result was not affected by any single study (S1–S4 Tables)

#### pTERTm and prognostic significance

**Fig 2:**
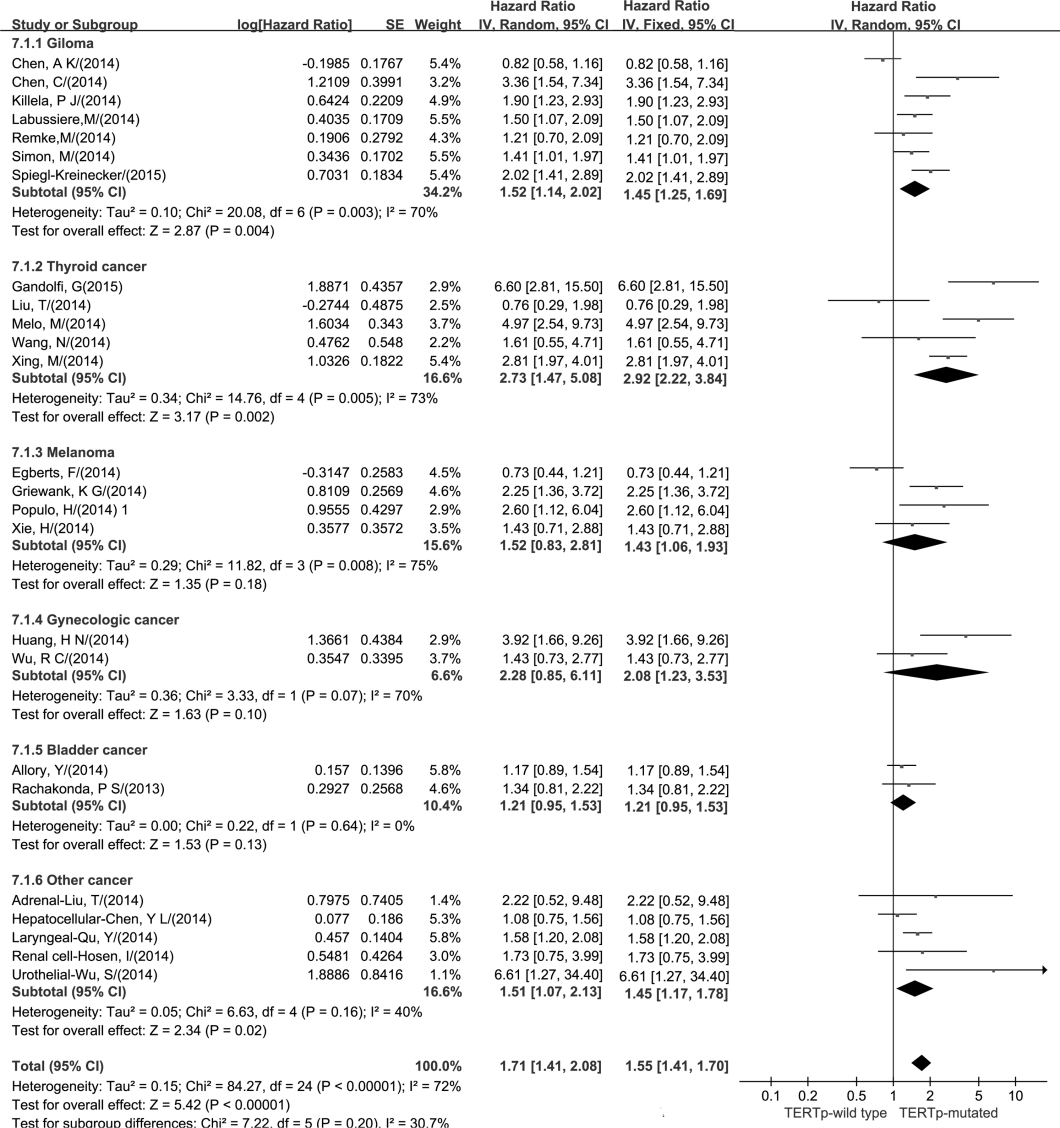
Hazard ratios (HRs) and 95% confidence intervals (95% CIs) for patient prognosis (5-year overall survival rate) associated with pTERTm (carriers vs. noncarriers). The random effect model and fixed effect model are both showed.

The HRs for 5-year overall survival were available from 25 studies. All of the studies were published between 2013 and 2015 and were carried out in China, Japan, Austria, the United States, Germany, France, Spain and Portugal. We found a significant increased risk of death for the pTERTm carriers (HR = 1.71; p <0.0001) (Tables 4 and 5). Stratification analysis identified significant risk in subgroups of glioma (HR = 1.52; p = 0.004), thyroid cancer (HR = 2.73; p = 0.002), gynecologic cancer (HR = 2.08; p = 0.006) and “other cancer” (HR = 1.45; p = 0.0005) (Fig 2, Table 4). All the results of the meta-analyses are showed in a simplified table (Table 5).

**Table 5:** Conclusion results of Meta-analyses.

	Effect model		
Analysis	Fixed	Random	P value
Age (MD, carriers vs. noncarriers)	- -	5.24 [2.00, 8.48]	0.002
Gender (OR, Male vs. Female)	1.38 [1.22, 1.58]	- -	<0.0001
LM (OR, positive vs. negative)ct	- -	1.02 [0.71, 1.46]	0.93
DM (OR, positive vs. negative)	- -	3.78 [2.45, 5.82]	<0.0001
Tumor stage (OR, III/IV vs. I/II)	- -	2.48 [1.48, 4.15]	0.0005
Glioma WHO grade (OR, III&IV vs. I/II)	2.41 [1.88, 3.08]	- -	<0.00001
Prognosis (HR, carriers vs. noncarriers)	- -	1.71 [1.41, 2.08]	<0.0001

OR: odds ratio; MD: mean difference; HR: hazard ratio; WHO: World Health Organization; LM: lymphatic metastasis; DM: distant metastasis

We preformed meta-regression analyses by covariates including population, sample size, age, treatment, HR estimation and NOS score. No significant alteration was found in the HR by these covariates, and the results showed that the differences between the subgroups did not reach statistical significance (Table 6). No evidence was found to demonstrate that any of these covariates could explain the heterogeneity. In addition, sensitivity analyses omitting one study each time showed that the study of Chen, A K (glioma), Liu, T (Thyroid cancer) and Egberts, F (Melanoma) had the largest influence on the result; The heterogeneity become non-significant when they are omitted. And the summary HR of melanoma became significant and heterogeneity disappeared when the study of Egberts, F was omitted (HR = 2.04; 95% CI = 1.41 to 2.95) (S5 Table).

**Table 6:** Results of meta-regression analyses in prognosis.

Factors and	No. of	No. of	Summary HR, 95% CI		Heterogeneity		Meta-regression
Subgroups	studies	patients	Fixed effect model	Random effect model	I^2^ (%)	p	P-value
**5-year overall survival**	**25**	**6415**	- -	**1.71 [1.41, 2.08]**	**72**	**<0.0001**	
Population							0.981
Asians	6	1167	- -	1.74 [1.09, 2.77]	80	0.0001	
Non-Asian	17	5013	- -	1.76 [1.40, 2.23]	71	0.0003	
Sample size							0.95
≥195	14	4004	- -	1.81 [1.42, 2.31]	78	0.0001	
<195	11	2411	- -	1.55 [1.11, 2.17]	56	0.01	
Age							0.329
≥54	13	2918	1.49 [1.27, 1.75]	- -	31	0.13	
<54	12	3497	- -	1.99 [1.36, 2.91]	82	<0.0001	
Treatment							0.654
Surgery alone	12	3130	1.58 [1.19, 2.08]	- -	59	0.005	
Combined	11	2884	- -	1.83 [1.34, 2.50]	82	<0.0001	
HR estimation							0.205
Reported/calculated	9	1488	1.40 [1.19, 1.64]	- -	0	0.51	
Estimate	16	4927	- -	1.94 [1.46, 2.57]	78	<0.0001	
Study quality score							0.79
≤7	12	3219	- -	1.61 [1.19, 2.17]	74	<0.0001	
>7	13	3196	- -	1.82 [1.40, 2.36]	69	0.0006	

HR: hazard ratio

#### Publication bias

Begg’s funnel plot and Egger’s test were both performed to evaluate the publication bias of the studies. The shapes of the funnel plots did not show any evidence of an obvious asymmetry in any comparison model. As shown in S5 Fig The p value of Egger’s regression tests further provided evidence of funnel plot symmetry. (Table 7).

**Table 7:** P values for Begg's funnel and Egger's test in meta-analysis.

Meta-Analysis	Egger's test
Age at diagnosis	0.108
Gender	0.516
Distant metastasis	0.643
Tumor stage	0.188
prognosis	0.062

## Discussion

The maintenance of telomere length is of ultimate importance to normal self-renewing stem cells and cancer cells for preventing senescence induction. It has been suggested that tumour cells rely on epigenetic mechanisms or alterations that maintain telomerase activity to retain their immortality [49,50,51]. The recurrent pTERTm creates a putative binding site for ETS/TCF binding motifs, thereby facilitating the transcription of TERT [7,8]. pTERTm have recently been shown as a novel genetic mechanism underlying telomerase activation and present in diverse human tumours with a large range of prevalence. It was first reported in the melanoma, and then the prevalence of pTERTm was reported in 43–51% of cancers of central nervous system, 59–66% of bladder, 59% of hepatocellular, 10% of thyroid cancer, and 29–73% of skin cancers. Nonetheless, pTERTm was found absent in breast carcinoma, low in cancers of digestive system organs, haematopoietic system and certain reproductive system (serous carcinoma)[52].

The prevalence of pTERTm in small-cell lung cancer (SCLC) and NSCLC have been investigated. Zheng et al [22] failed to detect presence of pTERTm in SCLC. Chen et al [21] and Li et al [20] tried to identify pTERTm in NSCLC but no positive result was found. However, in the present studies, we identified a low frequency of pTERTm (5.8%) in NSCLCs and the mutation was significantly associated with older patients, similar to the result of Ma and his colleagues [19]. They detected 8 adenocarcinomas, 3 squamous carcinoma and 1 other histologic type of 467 NSCLC patients are pTERTm carriers. we tried to further investigate the association of pTERTm with tumour size, differentiation level and distant metastasis, but no significant association was found.

In the current meta-analysis, a borderline significant association between pTERTm and relevant clinical data was observed in overall analysis except for lymphatic analysis. The obvious between-study heterogeneity in each analysis decreased markedly in stratification analyses by tumour types, suggesting that different tumour types might be a potential source of heterogeneity. Interestingly, we observed a significant association of pTERTm with a higher age at diagnosis in patients with glioma and thyroid cancer, whereas patients with melanoma displayed an opposite pattern. This is probably because genetic factors and environmental factors contribute equally to the development of melanoma. Recent studies suggested that melanoma is found more frequently in skin with intermittent sun-exposure than in skin that is not exposed or chronically exposed [53,54].

In addition, we found that thyroid cancer patients with pTERTm have a higher risk of distant metastasis that is four times greater than that of patients without pTERTm (OR = 4.01, 95% CI = 3.15 to 5.10), in line with the study done by Gandofi et al. They found that pTERTm are strongly associated with tumour progression and development of distant metastasis in papillary thyroid cancer [31]. Similarly, landa et al demonstrated that pTERTm are highly prevalent in advanced thyroid cancers (51%) compared to well-differentiated tumours (22%) [55]. Taken together, these data indicate that pTERTm is probably a genetic event during the acquisition of metastatic potential. The mechanism of pTERTm in cancer progression is still unclear. It has been reported that pTERTm is able to increase the transcriptional activity of TERT promoter in tumours and express higher level of TERT mRNA compared with wild type-tumours [7,8,11,33,39,56]. In this regard, it is conceivable that the acquisition of pTERTm leading to TERT activation is an important event during cancer progression, as it allows tumour cells to avoid proliferation limitation and to acquire immortalization [37]. Another study done by Papathomas et al reported that pTERTm occur preferentially in succinate dehydrogenase (SDH)-deficient tumours, and this genetic alteration might cooperate with pTERTm to extend the lifespan of mutated clones, so as to render them infinite proliferation potential and accumulation of additional genetic alterations [57]. However, such association was not found in melanoma, renal cell carcinoma and “other cancer”. Whether this effect may be cancer-type specific and play a different role in the etiology of other cancer are still unclear, thus the results should be interpreted with caution.

The 5-year overall survival data from 25 studies indicated that patients with pTERTm had a 70% greater risk of death than those without pTERTm. Since pTERTm results in the creation of binding sites for ETS/TCF transcription factors, which are downstream targets of RAS-RAF-MAPK pathways. pTERTm are suggested to have synergistic effects to promoter tumour cell proliferation with activating BRAF or NRAS mutations, which have been proposed to be driver mutations in the development of cutaneous melanocytic neoplasms. It is likely that these mutations turn the pTERTm into a target of ETS-domain transcription factors. Thus additional studies could further investigate whether pTERTm are of therapeutic significance, either in terms of influencing the efficacy of established therapies (ie, BRAF/NRAS inhibitors or immunotherapies) or whether they might even prove to be directly valuable to therapeutic targets[6,58,59]. The association between pTERTm and cancer prognosis was carefully investigated. We attempted to trace the origin of the substantial heterogeneity by performing subgroup and meta-regression analyses. Prognosis analyses in gynecologic cancer, bladder cancer and “other cancer” filed to exhibit significant heterogeneity when stratified by cancer types without changing the HR materially. Further Meta regression analysis by prespecified factors such as population, sample size, age, treatment, method of HR estimation and NOS score did not change the HR as well, and provide no evidence to account for the heterogeneity. In addition, the heterogeneity became non-significant in glioma, thyroid cancer and melanoma by sensitivity analysis.

The funnel plots and Egger’s test did not identify any publication bias. However, some limitations should be addressed in the interpretation of the results of our cohort study and meta-analysis. First, the sample size of our cohort study was relatively small. Well-designed population-based studies with large sample sizes and detailed exposure information are needed to further confirm our findings. Second, subgroup meta-analysis stratified by cancer type, such as hepatocellular carcinoma, bladder cancer and laryngeal cancer, might contain insufficient data to enforce statistical power to check for an association, despite our efforts to contact the authors for data. We were unable to include more articles because the authors of a few studies with incomplete data failed to reply to our requests. Hence, more individual study would be required to draw a more precise conclusion

In conclusion, we found that pTERTm is present in a small fraction of NSCLCs and are significantly associated with older patients. The meta-analyses suggested that pTERTm carriers were older than noncarriers in glioma, thyroid cancer and lung cancer, with melanoma demonstrate a reserved pattern. Male cancer patients exhibited a significantly elevated risk of having pTERTm in thyroid cancer, melanoma and hepatocellular carcinoma. Apart from other cancers, we also identified thyroid cancer patients with hTERTm are more likely to have distant metastasis and higher tumour stages. In addition, pTERTm carriers had a higher risk of death in our prognosis analysis in giloma, thyroid cancer, gynecologic cancer and “other cancers”. All in all, the detection of pTERTm appears to be a promising prognostic indicator in patients with cancer and may have potential as a biomarker for treatment stratification. More well-designed prospective studies are needed to validate our findings.

## Supporting Information

S1 PRISMA Checklist PRISMA 2009 Flow Diagram. (DOC)

S1 Fig Forest plot of meta-analysis of age at diagnosis associated with TERT promoter mutation (carriers vs. noncarriers). (TIF)

S2 Fig Forest plot of meta-analysis of patient gender associated with TERT promoter mutation. (TIF)

S3 Fig Forest plot of meta-analysis of distant metastasis in patient associated with TERT promoter mutation. (TIF)

S4 Fig Forest plot of meta-analysis of tumour stage of patient associated with TERT promoter mutation. (TIF)

S5 Fig Funnel plots to examine the possibility of publication bias in the data for age (A), gender (B), distant metastasis (C), tumour stage (D) and 5-year overall survival (E). (TIF)

S1 Table Sensitivity analyses of included studies in age analyses. (DOCX)

S2 Table Sensitivity analyses of included studies in gender analyses. (DOCX)

S3 Table Sensitivity analyses of included studies in distant metastasis. (DOCX)

S4 Table Sensitivity analyses of included studies in stage analyses. (DOCX)

S5 Table Sensitivity analyses of included studies in prognosis. (DOCX)
