# Supplementary figures and images for: Clinical Characteristics and Prognostic Significance of TERT Promoter Mutations in Cancer: A Cohort Study and a Meta-Analysis
Source: PLoS One. 2016 Jan 22;11(1):e0146803. doi: 10.1371/journal.pone.0146803 (PMC4723146; doi:10.1371/journal.pone.0146803)

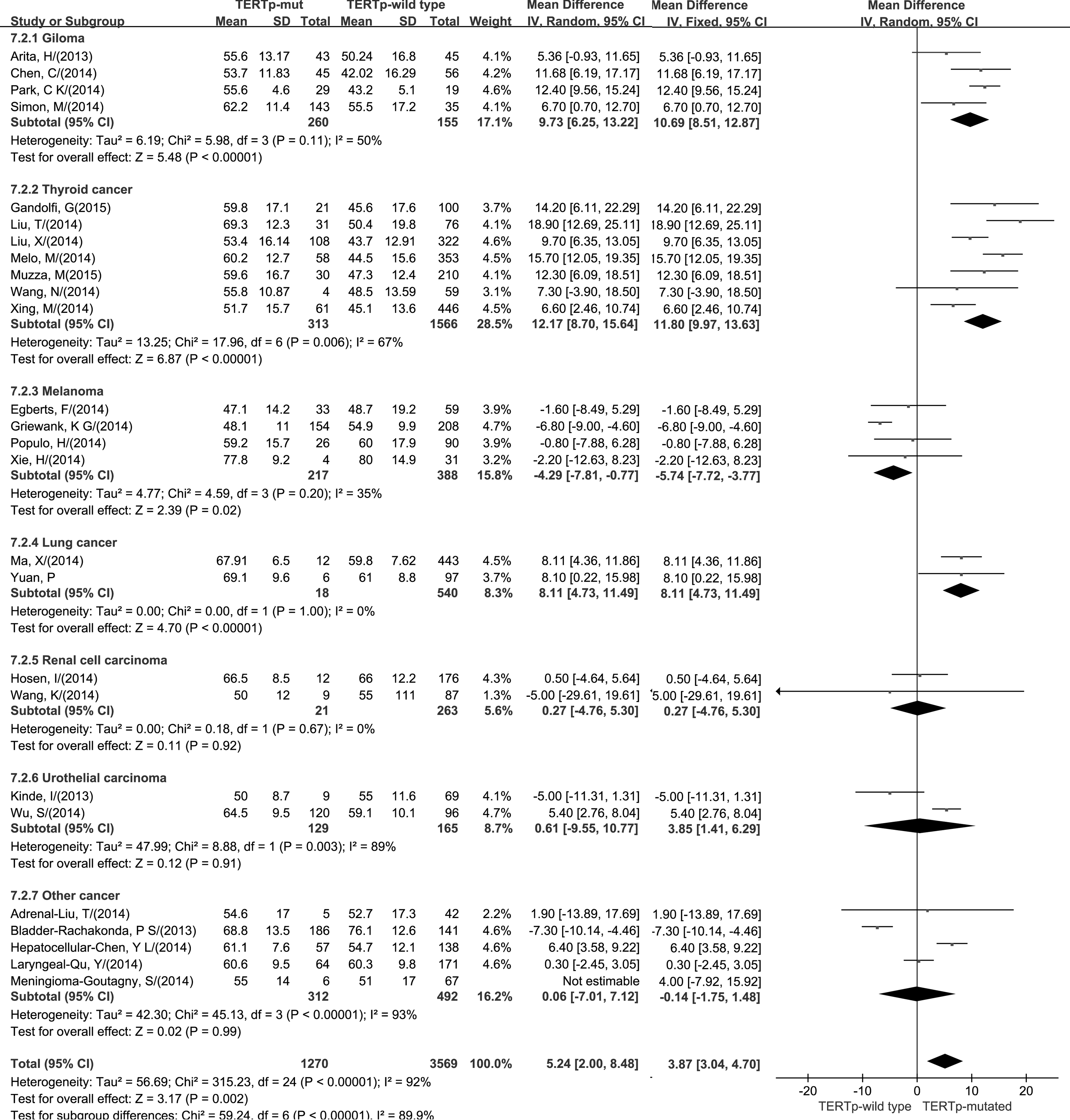

Supplement: S1 Fig — (TIF) [file pone.0146803.s002.tif]

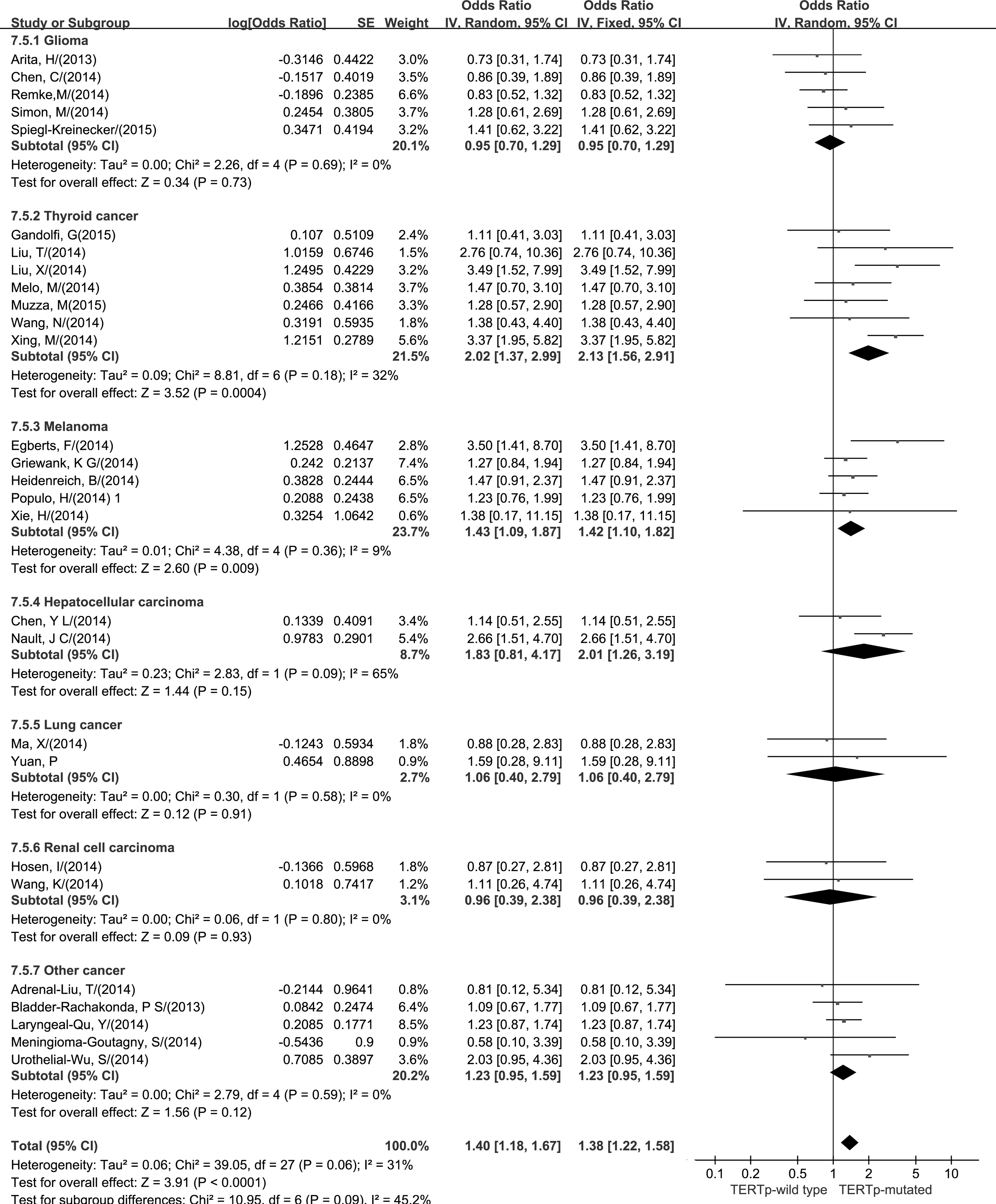

Supplement: S2 Fig — (TIF) [file pone.0146803.s003.tif]

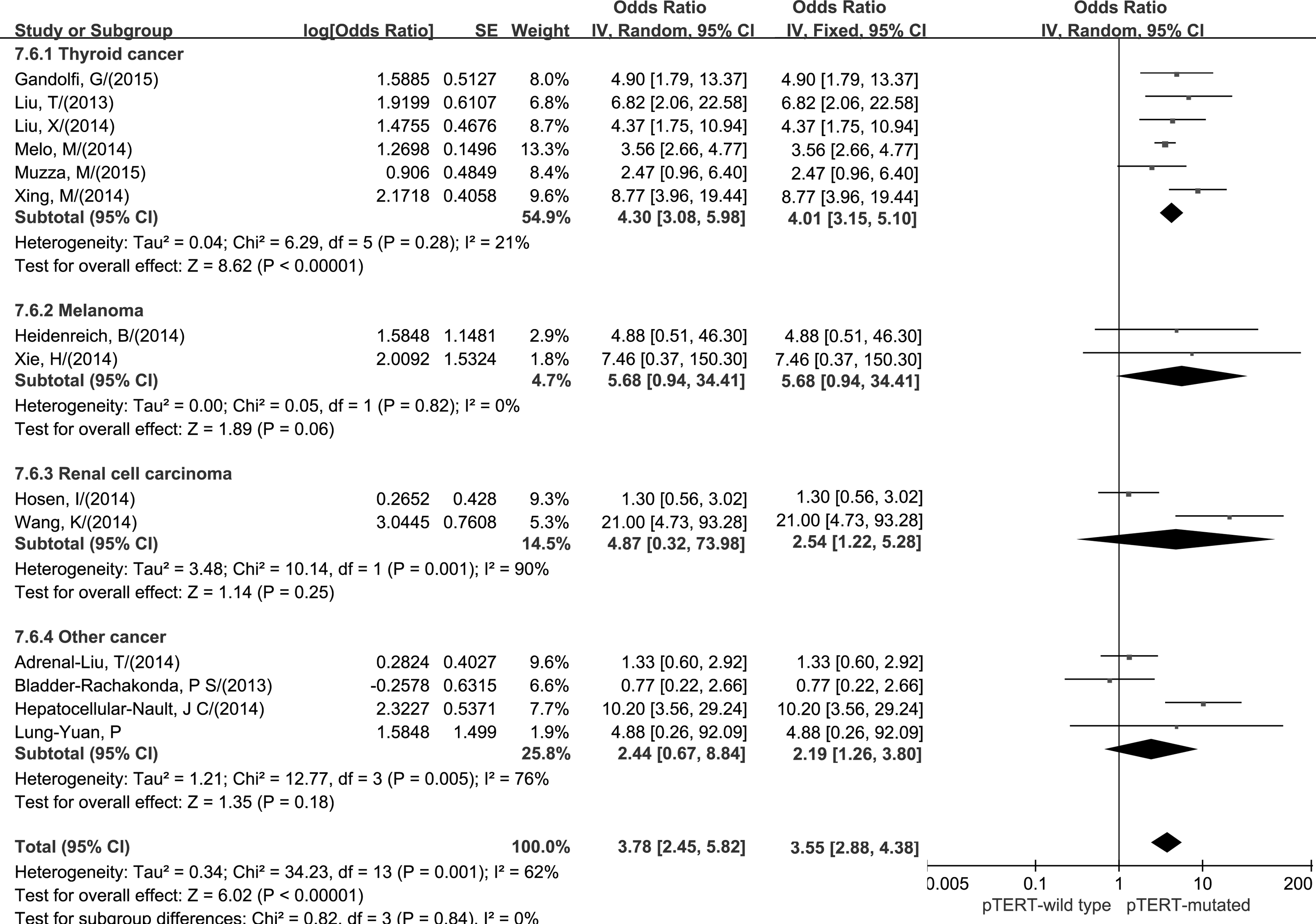

Supplement: S3 Fig — (TIF) [file pone.0146803.s004.tif]

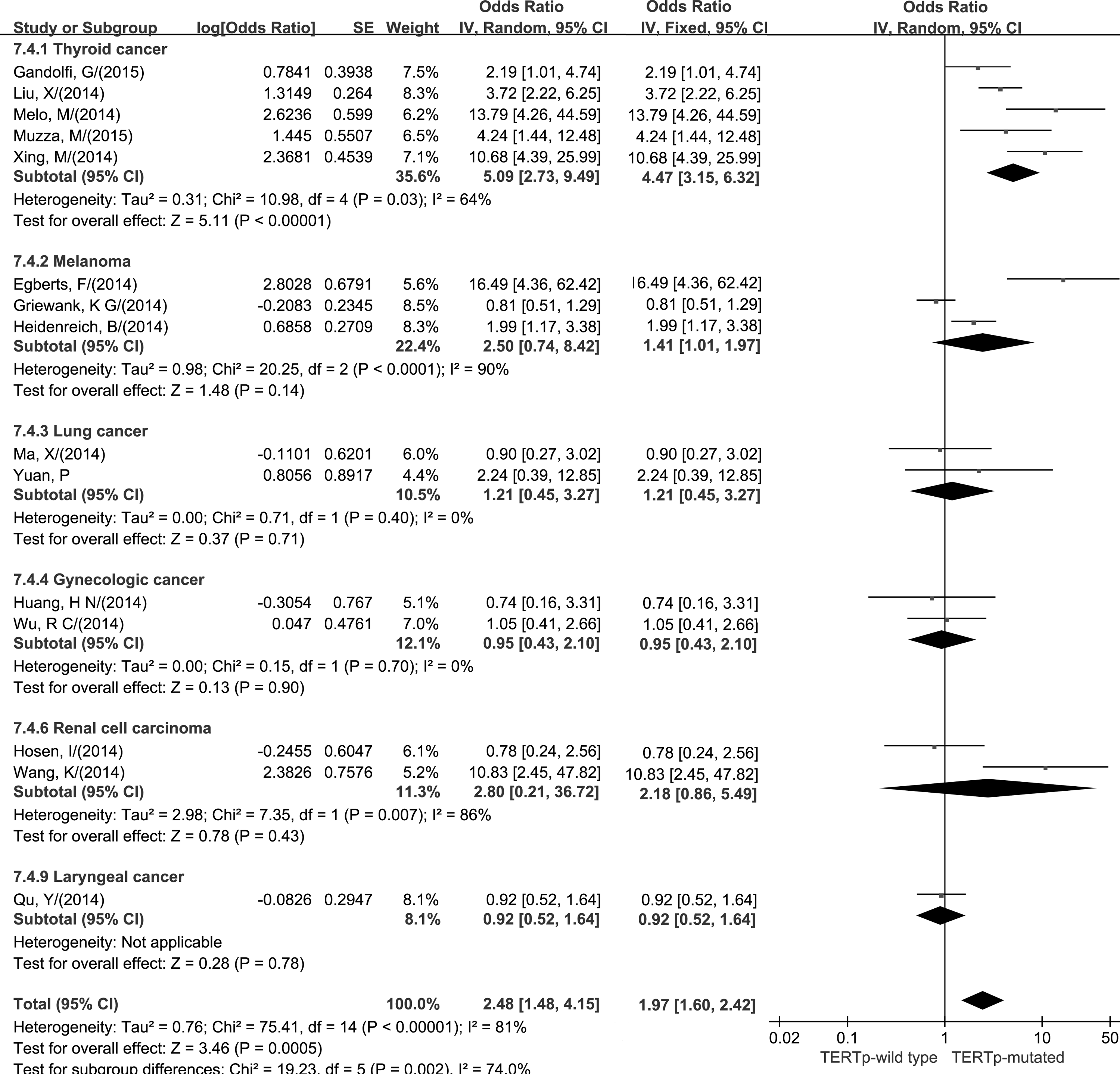

Supplement: S4 Fig — (TIF) [file pone.0146803.s005.tif]

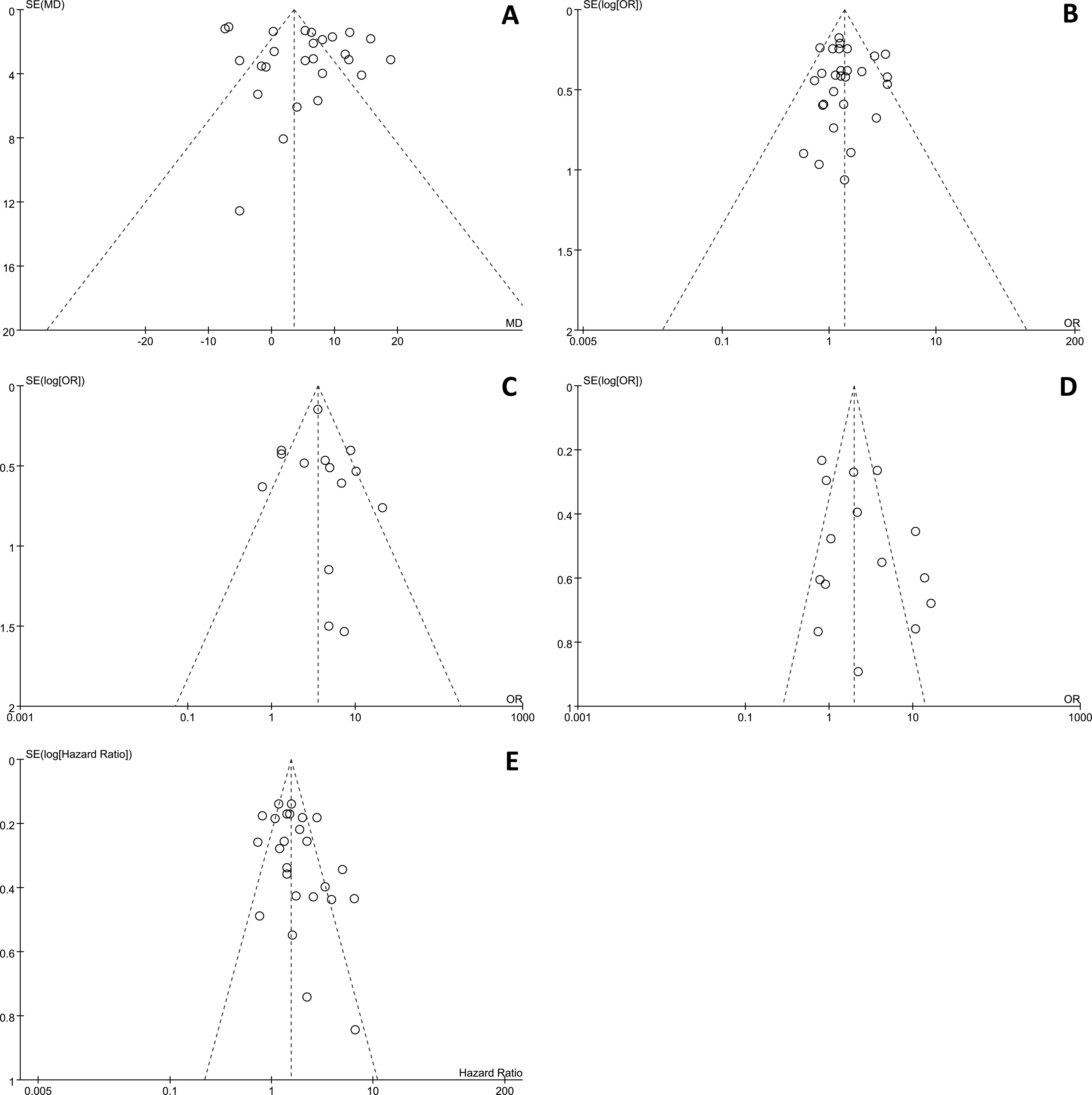

Supplement: S5 Fig — (TIF) [file pone.0146803.s006.tif]
