# Supplementary material for: Clinical Characteristics and Prognostic Significance of TERT Promoter Mutations in Cancer: A Cohort Study and a Meta-Analysis
Source: PLoS One. 2016 Jan 22;11(1):e0146803. doi: 10.1371/journal.pone.0146803 (PMC4723146; doi:10.1371/journal.pone.0146803)
Supplement: S1 Table — (DOCX) [file pone.0146803.s007.docx]

**S1 Table. Sensitivity analyses of included studies in age analyses**

|  |  |  |  |  |  |  |  |
| --- | --- | --- | --- | --- | --- | --- | --- |
|  |  |  | Sensitivity analysis in subgroup (when omitted) | | | |  |
|  |  | Carriers/ | Summary subgroup MD,95%CI | |  | Heterogeneity | |
|  | Study/year | noncarriers | Fixed | Random |  | I^2^ (%) | p |
| **Giloma** | |  |  |  |  |  |  |
|  | Arita, H/2013 | 43/45 | 11.41 [9.09, 13.74] | -- |  | 30 | 0.24 |
|  | Chen, C/2014 | 45/56 | 10.50 [8.12, 12.88] |  |  | 66 | 0.05 |
|  | ***Park, C K/2014*** | ***29/19*** | ***8.22 [4.82, 11.63]*** | ***--*** |  | ***22*** | ***0.28*** |
|  | Simon, M/2014 | 143/35 | 11.29 [8.95, 13.64] | -- |  | 50 | 0.13 |
| **Thyroid cancer** | |  |  |  |  |  |  |
|  | Gandolfi, G/2015 | 21/100 | -- | 11.94 [8.10, 15.77] |  | 72 | 0.003 |
|  | Liu, T/2014 | 31/76 | -- | 11.12 [7.78, 14.47] |  | 60 | 0.03 |
|  | Liu, X/2014 | 108/322 | -- | 12.75 [8.49, 17.01] |  | 68 | 0.005 |
|  | Melo, M/2014 | 58/353 | -- | 11.31 [7.65, 14.98] |  | 59 | 0.03 |
|  | Muzza, M/2015 | 30/210 | -- | 12.17 [8.15, 16.18] |  | 72 | 0.003 |
|  | Wang, N/2014 | 4/59 |  | 12.54 [8.86, 16.23] |  | 71 | 0.004 |
|  | ***Xing, M/2014*** | ***61/446*** | ***13.07 [11.03, 15.12]*** | ***--*** |  | ***52*** | ***0.06*** |
| **Melanoma** | |  |  |  |  |  |  |
|  | Egberts, F/2014 | 33/59 | -6.11 [-8.17, -4.06] | -- |  | 35 | 0.21 |
|  | ***Griewank, K G/2014*** | ***154/208*** | ***-1.39 [-5.85, 3.07]*** | ***--*** |  | ***0*** | ***0.97*** |
|  | Populo, H/2014 | 26/90 | -6.16 [-8.21, -4.11] | -- |  | 22 | 0.28 |
|  | Xie, H/2014 | 4/31 | -5.88 [-7.88, -3.87] | -- |  | 52 | 0.13 |
| **Lung cancer** | |  |  |  |  |  |  |
|  | Ma, X/2014 | 12/443 | -- | -- |  | -- | -- |
|  | Yuan, P | 6/97 | -- | -- |  | -- | -- |
| **Renal cell carcinoma** | |  |  |  |  |  |  |
|  | Hosen, I/2014 | 12/176 | -- | -- |  | -- | -- |
|  | Wang, K/2014 | 9/87 | -- | -- |  | -- | -- |
| **Urothelial carcinoma** | |  |  |  |  |  |  |
|  | Kinde, I/2013 | 9/69 | -- | -- |  | -- | -- |
|  | Wu, S/2014 | 120/96 | -- | -- |  | -- | -- |
| **Other cancer** | |  |  |  |  |  |  |
|  | Adrenal-Liu, T/2014 | 5/42 | -- | 0.45 [-6.67, 7.58] |  | 90 | <0.001 |
|  | ***Bladder-Rachakonda, P S/2013*** | ***186/141*** | ***--*** | ***3.31 [-1.25, 7.87]*** |  | ***66*** | ***0.03*** |
|  | Hepatocellular-Chen, Y L/2014 | 57/138 | -- | -1.75 [-7.77, 4.27] |  | 81 | <0.001 |
|  | Laryngeal-Qu, Y/2014 | 64/171 | -- | 0.91 [-8.84, 10.66] |  | 90 | <0.001 |
|  | Meningiomas-Goutagny, S/2014 | 6/67 | -- | 0.06 [-7.01, 7.12] |  | 90 | <0.001 |
|  | | | | |  |  |  |

**SMD: standardised mean difference; Studies with the largest influence are bold and italic**
