# Supplementary material for: Clinical Characteristics and Prognostic Significance of TERT Promoter Mutations in Cancer: A Cohort Study and a Meta-Analysis
Source: PLoS One. 2016 Jan 22;11(1):e0146803. doi: 10.1371/journal.pone.0146803 (PMC4723146; doi:10.1371/journal.pone.0146803)
Supplement: S2 Table — (DOCX) [file pone.0146803.s008.docx]

**S2 Table. Sensitivity analyses of included studies in gender analyses**

|  |  |  |  |  |  |  |
| --- | --- | --- | --- | --- | --- | --- |
|  |  |  | Overall sensitivity analysis (when omitted) | | | |
|  |  | Carriers/ | Summary subgroup OR, 95% CI |  | Heterogeneity | |
|  | Study/year | noncarriers | Fixed effect model |  | I^2^ (%) | p |
| **Giloma** | |  |  |  |  |  |
|  | Spiegl-Kreinecker/2015 | 92/34 | 0.89 [0.64, 1.23] |  | 0 | 0.75 |
|  | Simon, M/2014 | 141/35 | 0.89 [0.64, 1.25] |  | 0 | 0.68 |
|  | Remke,M/2014 | 93/365 | 1.05 [0.70, 1.56] |  | 0 | 0.53 |
|  | Chen, C/2014 | 45/101 | 0.96 [0.69, 1.34] |  | 0 | 0.54 |
|  | Arita, H/2013 | 43/45 | 0.98 [0.71, 1.36] |  | 0 | 0.6 |
| **Thyroid cancer** | |  |  |  |  |  |
|  | ***Xing, M/2014*** | ***142/446*** | ***1.71 [1.18, 2.50]*** |  | ***0*** | ***0.44*** |
|  | Wang, N/2014 | 4/76 | 2.20 [1.60, 3.04] |  | 39 | 0.14 |
|  | Muzza, M/2015 | 30/210 | 2.32 [1.66, 3.25] |  | 29 | 0.22 |
|  | Melo, M/2014 | 58/411 | 2.30 [1.64, 3.24] |  | 39 | 0.18 |
|  | Liu, X/2014 | 42/325 | 1.97 [1.41, 2.75] |  | 31 | 0.2 |
|  | Liu, T/2014 | 31/76 | 2.10 [1.53, 2.89] |  | 42 | 0.12 |
|  | Gandolfi, G/2015 | 21/100 | 2.28 [1.65, 3.16] |  | 29 | 0.22 |
| **Melanoma** | |  |  |  |  |  |
|  | Xie, H/2014 | 4/35 | 1.42 [1.10, 1.83] |  | 32 | 0.22 |
|  | Populo, H/2014 | 102/298 | 1.50 [1.11, 2.01] |  | 24 | 0.27 |
|  | Heidenreich, B/2014 | 109/178 | 1.40 [1.04, 1.88] |  | 31 | 0.23 |
|  | Griewank, K G/2014 | 154/208 | 1.51 [1.10, 2.06] |  | 25 | 0.26 |
|  | ***Egberts, F/2014*** | ***33/59*** | ***1.32 [1.01, 1.71]*** |  | ***0*** | ***0.96*** |
| **Hepatocellular carcinoma** | |  |  |  |  |  |
|  | Chen, Y L/2014 | 57/138 | -- |  | -- | -- |
|  | Nault, J C/2014 | 179/126 | -- |  | -- | -- |
| **Renal cell carcinoma** | |  |  |  |  |  |
|  | Hosen, I/2014 | 12/176 | -- |  | -- | -- |
|  | Wang, K/2014 | 9/87 | -- |  | -- | -- |
| **Lung cancer** | |  |  |  |  |  |
|  | Ma, X | 12/455 | -- |  | -- | -- |
|  | Yuan, P | 6/97 | -- |  | -- | -- |
| **Other cancer** | |  |  |  |  |  |
|  | Adrenal-Liu, T/2014 | 5/42 | 1.24 [0.95, 1.61] |  | 57 | 0.46 |
|  | Bladder-Rachakonda, P S/2013 | 186/93 | 1.29 [0.95, 1.75] |  | 40 | 0.48 |
|  | Laryngeal-Qu,Y/2014 | 64/171 | 1.23 [0.83, 1.81] |  | 40 | 0.43 |
|  | Meningioma-Goutagny, S/2014 | 6/67 | 1.25 [0.96, 1.62] |  | 39 | 0.56 |
|  | ***Urothelial -Wu, S/2014*** | ***120/96*** | ***1.15 [0.87, 1.52]*** |  | ***38*** | ***0.82*** |
|  | | | |  |  |  |

**NR: No report. Studies with the largest influence are bold and italic**
