# Supplementary material for: Clinical Characteristics and Prognostic Significance of TERT Promoter Mutations in Cancer: A Cohort Study and a Meta-Analysis
Source: PLoS One. 2016 Jan 22;11(1):e0146803. doi: 10.1371/journal.pone.0146803 (PMC4723146; doi:10.1371/journal.pone.0146803)
Supplement: S3 Table — (DOCX) [file pone.0146803.s009.docx]

**S3 Table. Sensitivity analyses of included studies in distant metastasis**

|  |  |  |  |  |  |  |  |
| --- | --- | --- | --- | --- | --- | --- | --- |
|  |  |  | sensitivity analysis (when omitted) | | | | |
|  |  | Carriers/ | Summary subgroup OR, 95% CI | |  | Heterogeneity | |
|  | Study/year | noncarriers | Fixed effect model | Random effect model |  | I^2^ (%) | p |
| **Thyroid cancer** | |  |  |  |  |  |  |
|  | Gandolfi, G/2015 | 21/100 | 3.96 [3.09, 5.08] | -- |  | 35 | 0.19 |
|  | Liu, T/2014 | 31/76 | 3.92 [3.06, 5.01] | -- |  | 27 | 0.24 |
|  | Liu, X/2014 | 39/369 | 3.98 [3.10, 5.11] | -- |  | 36 | 0.18 |
|  | Melo, M/2014 | 58/411 | 5.13 [3.36, 7.84] | -- |  | 8 | 0.36 |
|  | Muzza, M/2015 | 30/210 | 4.14 [3.23, 5.32] | -- |  | 24 | 0.26 |
|  | ***Xing, M/2014*** | ***61/446*** | ***3.70 [2.87, 4.77]*** | ***--*** |  | ***0*** | ***0.7*** |
| **Melanoma** | |  |  |  |  |  |  |
|  | Heidenreich, B/2014 | 109/178 | -- | -- |  | -- | -- |
|  | Xie, H/2014 | 4/35 | -- | -- |  | -- | -- |
| **Renal cell carcinoma** | |  |  |  |  |  |  |
|  | Hosen, I/2014 | 12/176 | -- | -- |  | -- | -- |
|  | Wang, K/2014 | 9/87 | -- | -- |  | -- | -- |
| **Other** | |  |  |  |  |  |  |
|  | Adrenal-Liu, T/2014 | 5/42 | -- | 3.24 [0.46, 22.68] |  | 57 | 0.006 |
|  | Bladder-Rachakonda, P S/2013 | 186/93 | -- | 3.76 [0.74, 18.94] |  | 79 | 0.009 |
|  | ***Hepatocellular-Nault, J C/2014*** | ***179/126*** | ***1.22 [0.64, 2.33]*** | ***--*** |  | ***0*** | ***0.49*** |
|  | Lung-Yuan, P | 6/86 | -- | 2.20 [0.51, 9.53] |  | 84 | 0.002 |
|  | | | |  |  |  |  |

**OR: odds ratio. Studies with the largest influence are bold and italic**
