# Supplementary material for: Clinical Characteristics and Prognostic Significance of TERT Promoter Mutations in Cancer: A Cohort Study and a Meta-Analysis
Source: PLoS One. 2016 Jan 22;11(1):e0146803. doi: 10.1371/journal.pone.0146803 (PMC4723146; doi:10.1371/journal.pone.0146803)
Supplement: S4 Table — (DOCX) [file pone.0146803.s010.docx]

**S4 Table. Sensitivity analyses of included studies in stage analyses**

|  |  | |  |  |  |  |  |  |  |
| --- | --- | --- | --- | --- | --- | --- | --- | --- | --- |
|  |  | |  |  | Sensitivity analysis in subgroup (when omitted) | | | | |
|  |  | | carriers/ |  | Summary subgroup OR,95%CI | |  | Heterogeneity | |
|  | Study/year | | noncarriers |  | Fixed effect model | Random effect model |  | I^2^ (%) | p |
| **Thyroid cancer** | | | 608/2756 |  |  |  |  |  |  |
|  | | Gandolfi, G/2015 | 21/100 |  | 5.36 [3.63, 7.91] | -- |  | 56 | 0.08 |
|  | | Liu, X/2014 | 26/213 |  |  | 5.82 [2.40, 14.09] |  | 70 | 0.02 |
|  | | Melo, M/2014 | 38/244 |  | 4.01 [2.78, 5.77] |  |  | 58 | 0.07 |
|  | | Muzza, M/2015 | 30/210 |  |  | 5.38 [2.50, 11.55] |  | 73 | 0.01 |
|  | | ***Xing, M/2014*** | ***61/446*** |  | ***3.82 [2.61, 5.57]*** |  |  | ***55*** | ***0.08*** |
| **Melanoma** | | |  |  |  |  |  |  |  |
|  | ***Egberts, F/2014*** | | ***46/32*** |  | ***--*** | ***1.26 [0.52, 3.02]*** |  | ***84*** | ***0.02*** |
|  | Griewank, K G/2014 | | 138/159 |  | -- | 5.22 [0.66, 41.25] |  | 88 | 0.004 |
|  | Heidenreich, B/2014 | | 107/174 |  | -- | 3.42 [0.18, 65.21] |  | 94 | <0.0001 |
| **Lung cancer** | | |  |  |  |  |  |  |  |
|  | Ma, X | | 12/455 |  | -- | -- |  | -- | -- |
|  | Yuan, P | | 6/97 |  | -- | -- |  | -- | -- |
| **Gynecologic cancer** | | |  |  |  |  |  |  |  |
|  | Huang, H N/2014 | | 9/47 |  | -- | -- |  | -- | -- |
|  | Wu, R C/2014 | | 29/146 |  | -- | -- |  | -- | -- |
| **Renal cell carcinoma** | | |  |  |  |  |  |  |  |
|  | Hosen, I/2014 | | 12/176 |  | -- | -- |  | -- | -- |
|  | Wang, K/2014 | | 9/87 |  | -- | -- |  | -- | -- |
| **Laryngeal cancer** | | |  |  |  |  |  |  |  |
|  | Qu, Y/2014 | | 64/170 |  | -- | -- |  | -- | -- |

| **OR: odds ratio; NR: no report; Studies with the largest influence are bold and italic** |  |  |  |
| --- | --- | --- | --- |
