# Supplementary material for: Clinical Characteristics and Prognostic Significance of TERT Promoter Mutations in Cancer: A Cohort Study and a Meta-Analysis
Source: PLoS One. 2016 Jan 22;11(1):e0146803. doi: 10.1371/journal.pone.0146803 (PMC4723146; doi:10.1371/journal.pone.0146803)
Supplement: S5 Table — (DOCX) [file pone.0146803.s011.docx]

**S5 Table. Sensitivity analyses of included studies in prognosis**

|  |  |  |  |  |  |  |  |
| --- | --- | --- | --- | --- | --- | --- | --- |
|  |  |  | Sensitivity analysis in subgroup (when omited) | | | | |
|  |  | HR | Summary subgroup HR,95%CI | |  | Heterogeneity | |
|  | Study/year | estimation | Fixed effect model | Random effect model |  | I^2^ (%) | p |
| **Giloma** | |  |  |  |  |  |  |
|  | Spiegl-Kreinecker/2015 | E | -- | 1.44 [1.05, 1.97] |  | 69 | 0.006 |
|  | Simon, M/2014 | C | -- | 1.56 [1.09, 2.21] |  | 75 | 0.001 |
|  | Remke,M/2014 | R | -- | 1.57 [1.14, 2.17] |  | 75 | 0.001 |
|  | Labussiere,M/2014 | E | -- | 1.54 [1.08, 2.19] |  | 75 | 0.001 |
|  | Killela, P J/2014 | E | -- | 1.47 [1.06, 2.03] |  | 73 | 0.001 |
|  | Chen, C/2014 | E | -- | 1.42 [1.08, 1.86] |  | 68 | 0.008 |
|  | ***Chen, A K/2014*** | ***E*** | ***1.66 [1.40, 1.96]*** | ***--*** |  | ***31*** | ***0.21*** |
| **Thyroid cancer** | |  |  |  |  |  |  |
|  | Xing, M/2014 | E | -- | 2.62 [1.00, 6.88] |  | 80 | 0.002 |
|  | Wang, N/2014 | E | -- | 3.00 [1.48, 6.11] |  | 78 | 0.004 |
|  | Melo, M/2014 | E | -- | 2.29 [1.07, 4.92] |  | 75 | 0.008 |
|  | ***Liu, T/2014*** | ***C*** | ***3.29 [2.47, 4.38]*** | ***--*** |  | ***54*** | ***0.09*** |
|  | Gandolfi, G/2015 | E |  | 2.24 [1.14, 4.39] |  | 72 | 0.01 |
| **Melanoma** | |  |  |  |  |  |  |
|  | Xie, H/2014 | C | -- | 1.57 [0.68, 3.63] |  | 83 | 0.003 |
|  | Populo, H/2014 | C | -- | 1.33 [0.65, 2.70] |  | 79 | 0.008 |
|  | Griewank, K G/2014 | E | -- | 1.31 [0.63, 2.73] |  | 72 | 0.03 |
|  | ***Egberts, F/2014*** | ***E*** | ***2.04 [1.41, 2.95]*** | ***--*** |  | ***0*** | ***0.48*** |
| **Bladder cancer** | |  |  |  |  |  |  |
|  | Rachakonda, P S/2013 | E | -- | -- |  | -- | -- |
|  | Allory, Y/2014 | E | -- | -- |  | -- | -- |
| **Gynecology cancer** | |  |  |  |  |  |  |
|  | Huang, H N/2014 | E | -- | -- |  | -- | -- |
|  | Wu, R C/2014 | E | -- | -- |  | -- | -- |
| **Other cancer** | |  |  |  |  |  |  |
|  | Urothelial-Wu, S/2014 | E | 1.41 [1.14, 1.74] | -- |  | 9 | 0.35 |
|  | Renal cell-Hosen, I/2014 | C | 1.43 [1.15, 1.77] | -- |  | 53 | 0.09 |
|  | Laryngeal-Qu, Y/2014 | C | 1.28 [0.93, 1.77] | -- |  | 47 | 0.13 |
|  | ***Hepatocellular-Chen, Y L/2014*** | ***R*** | ***1.67 [1.29, 2.15]*** | ***--*** |  | ***0*** | ***0.39*** |
|  | Adrenal-Liu, T/2014 | C | 1.43 [1.16, 1.77] | -- |  | 52 | 0.1 |
|  |  | | | | | | |
|  |  | | | | | | |

**Treatment: S-surgery, R-radiotherapy, C-Chemotherapy; NR: no report. HR: hazard ratio;**

**HR estimation: R-reported, C-calculated, E-estimated; Studies with the largest influence are bold and italic**
